# Diversity Climate, Salutogenic Theory, and the Occupational Health of College-Educated Women from Conservative Communities

**DOI:** 10.3390/ijerph19042356

**Published:** 2022-02-18

**Authors:** Tehila Kalagy, Sarah Abu-Kaf, Nirit Portughies, Orna Braun-Lewensohn

**Affiliations:** 1 Department of Public Policy & Administration, Ben-Gurion University of the Negev, Beer-Sheva P.O. Box 653, Israel; 2 Conflict Management & Resolution Program, Department of Multidisciplinary Studies, Ben-Gurion University of the Negev, Beer-Sheva P.O. Box 653, Israel; aks@bgu.ac.il (S.A.-K.); portughies.nirit@gmail.com (N.P.); ornabl@bgu.ac.il (O.B.-L.)

**Keywords:** diversity climate, salutogenic theory, occupational health, workforce management, traditional college-educated women

## Abstract

Over the past four decades, there have been significant changes in workplaces around the world, including a workforce that has become more diverse as the relative proportion of women in the workforce has increased. This trend has included the increased workforce participation of women from conservative minority groups. This article discusses the significance of the integration of college-educated women from conservative minority groups into the workforce in terms of their own personal health and well-being. This work focuses on two groups of college-educated women from conservative minority groups that have joined the Israeli workforce: Ultra-Orthodox women and Bedouin Arab women. This qualitative study was based on five focus groups, which included 16 women from the two examined groups. The main themes raised in those focus groups were categorized and analyzed. The data analysis was guided by the diversity-climate approach and salutogenic theory. The research findings indicate that a diversity climate that included most of the different aspects of this approach was present in the participants’ statements regarding their workplaces. In practice, diversity climate supported sense of coherence, such that both diversity climate and a sense of coherence led directly to the occupational health of these college-educated, minority women.

## 1. Introduction

Over the past four decades, there have been significant changes in workplaces around the world. These have included changes in organizational structure, globalization processes, academic accreditations, the use of information technologies at work, and a workforce that has grown increasingly diverse with the increased participation of women, in general, and women from conservative minority groups in particular. The ideology of multiculturalism has also influenced perceptions regarding the acceptance of minorities and their position in society, as well as the development of a range of different approaches for making that happen. One well-known approach is that of diversity management [1,2], which involves recognizing the diversity of the workforce and the need to take into consideration the cultures from which individuals come. According to the research literature, successful diversity management can improve workers’ performance and well-being and lead to the economic growth of the organization. In this paper, we will discuss the significance of employment for the personal health and well-being of college-educated women who are members of minority groups. This work focuses on two groups of college-educated women from conservative minority groups who have integrated into the Israeli workforce: Ultra-Orthodox women and Bedouin Arab women. This work is particularly important as no such comparative study has been conducted previously. This comparison may provide support for theories regarding change processes in conservative minority communities, in general, and among women from these two populations, in particular. The need for this research is underscored by the demographic growth of both study populations and the need to optimally integrate these women into the workforce.

As of the start of 2021, there were 9.291 million residents of Israel, with several distinct sub-societies: secular Jews, religious Jews, Ultra-Orthodox Jews, and Arab Israelis, of which there are several different groups, including the Bedouin and Druze. The Arab population and the Ultra-Orthodox population are both expected to grow over the next 30 years. Today, Arabs account for 21% of the Israeli population, with Bedouin Arabs accounting for 3% of the Israeli population and 17% of the Arab minority. Ultra-Orthodox Jews account for 12% of the general Israeli population [3,4].

These two communities are struggling socioculturally, politically (mainly in the case of the Bedouin Arab community), and economically due to a lack of opportunities and/or inequality, problems that have different roots in the different communities [5]. The predicted demographic growth, together with low employment rates among Bedouin Arab women and Ultra-Orthodox men, has given rise to fears of hindered growth and increased poverty in these communities and Israeli society in general. Following the recommendations of governmental committees, national employment goals have been updated, including the goals for particular subgroups. In recent years, different programs for these minority groups have been developed to promote their integration into the Israeli labor market [6].

In this work, we have chosen to focus on women who are members of minority groups because they have the primary responsibility for the care and well-being of the family unit and, at the same time, are indicative of the family-level and societal-level changes that their communities are currently undergoing. These processes of change are related to women entering the world of higher education and their desire to find work that corresponds to their field of study. The process of Bedouin Arab and Ultra-Orthodox women exiting the borders of their communities and entering the general job market involves significant coping on both the practical level and the emotional level [7]. The movement between a non-Western, traditional society with religious social norms and a liberal, non-religious environment and the entry, as educated women, into a world that still includes glass ceilings [8] that limit occupational advancement present these women with challenges and unique conflicts at work and in their lives in general.

The vast majority of the research in this field has focused on barriers to the integration of individuals from minority groups into the job market. In this study, we wanted to focus specifically on factors that promote the wellbeing and occupational health of college-educated Bedouin Arab and Ultra-Orthodox women in the labor market from their point of view. This research is based on two approaches: salutogenic theory and diversity climate. These ideas are part of a proactive, positive health trend that has also been studied in the context of organizations [9]. They attempt to identify factors that preserve and promote personal, social, and environmental resources, to allow those integrating into an organization to cope with the challenges in their environments in a healthy manner [10,11,12].

### 1.1. Diversity Climate and Occupational Health

Strategies for managing diversity span a continuum that includes doing nothing at one end and goes on to include the enactment of laws prohibiting discrimination, the adoption of a comprehensive strategy for addressing diversity, and the taking of proactive steps to promote a diverse workforce [13,14]. Organizations that take the latter approach act in the spirit of a diversity climate. Although there is no one metric for assessing diversity climate, it is customary to view it as an organizational strategy that provides appropriate and equal opportunities to all workers and pursues the goal of integration through the advancement and preservation of diversity [15,16,17,18]. This makes for an inclusive, effective, competitive, and productive organization [19] that promotes the well-being of its workers [20].

From a productivity perspective, organizations that have this type of climate encourage communication between group members from different backgrounds, which encourages the expression of a variety of perspectives regarding how to cope with different problems. This increases productivity at the collective level, as well as the potential for innovation and prosperity [1]. When members of a cross-functional and culturally diverse team begin to work together, the differences between them, the referral to stereotypes, and the differences of opinion between team members may be quite stark. Over time, team members will begin to share information related to tasks and focus less on stereotypes, lessening the separatist climate, to successfully manage conflict and reach solutions. Cooperation and the sharing of information also build mutual trust and understanding. In such teams, everyone feels included and that their own uniqueness is respected [21].

In terms of workers’ health, diversity climate is a conceptual environmental resource [22] that helps workers to strengthen their own emotional resources (e.g., hope, optimism, sense of personal capability, and resilience), as they can be themselves and express their own unique perspectives, participate in decision-making processes that affect the organization, suggest innovative solutions, and feel that they are essential [15]. When personal resources are empowered, the individual is in the best position for coping with pressures and stressors. Such resources are a source of tremendous energy and motivation to cope with work demands [23]. Since individuals spend most of their time at work, these resources and positive experiences became an inseparable part of their general health [24].

Management that advocates diversity respects the different viewpoints and ideas—including those that conflict with one another—of all staff members and communicates in a manner that is open, shares information, is accessible, and expresses awareness and concern for everyone’s needs, expectations, and feelings [25]. Workers’ commitment to tasks increases because they feel more comfortable suggesting initiatives and striving to carry them out. They view the management of diversity as a source of great emotional, cognitive, and physical motivation [26]. Ultra-Orthodox women employed in heterogeneous workplaces consistently described their workplaces as respectful, inclusive, and providing opportunities. According to those women, their managers and colleagues expressed openness and invested noticeable resources in order to accommodate their religious and social needs [27].

In this work, we chose to analyze the statements of participants in light of the parameters defined by Mor Barak, Cherin, and Berkman [28] for a diversity climate. Those parameters are as follows: (a) an organization’s policies and management practices that affect members of minority groups and women, such as discrimination or preferential treatment in hiring and promotion; (b) management activities that can lead to the inclusion or exclusion of members of minority groups and women; (c) individuals’ perceptions regarding the importance of diversity in work groups and the organization as a whole; and (d) the level of comfort felt in interactions with members of other groups.

### 1.2. Salutogenic Theory and the Promotion of Employees’ Health

The salutogenic model positions the health of an individual at any given moment on a continuum between comfort and discomfort and views health as the active, functional, and successful coping with the stresses of life [29,30]. Antonovsky [30], who studied how people cope with stress, believed that salutogenesis and the concept that lies at its heart, sense of coherence (SOC), are fundamental for coping in any cultural context.

The goal of SOC is the evaluation of the question: To what extent do individuals know how to cope with the difficult world that surrounds them? Antonovsky maintained that, over the course of their lives, individuals frequently encounter and must deal with stressful events and irritations. Based on that assumption, Antonovsky [31] defined health as a function of the interaction between the individual and human and other systems in his or her environment at any given time.

SOC is an individual-level construct that has cognitive, emotional, and behavioral components [31]. It is a global orientation that is expressed as the degree to which an individual perceives the world as comprehensible, manageable (i.e., the individual can cope with and affect reality), and meaningful. In accordance with the salutogenic model, SOC has an important influence on the health of the individual [30]. The unique nature of this concept can be seen in how it applies across borders and individual differences, such as culture, gender, age, and socioeconomic status. In his work, Antonovsky attempted, among other things, to identify the micro- and macro-level social conditions that encourage high levels of SOC [10]. Individuals with high levels of personal SOC view environmental pressures as challenges. They believe that the events that they experience can be explained and managed [29], and they do not feel as if they are victims of life events. These feelings and perceptions affect their health and performance [30,32].

### 1.3. Theoretical Integration

Diversity climate and the theory of salutogenesis are related to one another and may be seen as complementary to each other concerning integration into an employment context [33]. The resources provided by work, as a result of a diverse organizational culture, are expected to strengthen and structure an individual’s SOC [34].

Feldt, Kinnenen, and Mauno [35] found a strong relationship between SOC and organizational climate. Specifically, they found that employees who perceived their organizational climate as positive and felt that they had a high level of job security reported higher SOC scores. A positive climate helps individuals to see their environment as meaningful. Those researchers also found that SOC was diminished as the organizational climate became more negative. High levels of SOC have been associated with positive climates among university staff members [36] and within families [37]. Climates seen as contributing to the experience of meaningfulness in life and regard for life have also been associated with high levels of SOC [38].

### 1.4. Cultural Communities Examined in This Study

The process by which Ultra-Orthodox and Bedouin Arab women enter the general workforce involves significant coping on both the practical level and the emotional level. These challenges may place these women in positions of ongoing conflict in their workplaces. From a social perspective, employers and colleagues present these women with opportunities for emotional and social interactions with people with whom they might not otherwise have come into contact [39,40].

### 1.5. The Integration of Ultra-Orthodox Women into the Israeli Workforce

In recent decades, Ultra-Orthodox society (in all of its diversity) has undergone processes of comprehensive and fundamental cultural change. From an economic perspective, the contrast between the increased standard of living among the dominant society and the economic distress found among the Ultra-Orthodox has affected the economy of the family unit [41]. From a cultural perspective, the increased need for professional training and higher education, in light of women going out into the workforce and the reduced possibilities for making a livelihood in the field of education, have affected the conduct of the family unit [42]. In the Ultra-Orthodox community, most women are their family’s primary breadwinners, as their husbands dedicate themselves to religious study. For this reason, and particularly in the last decade, there has been an accelerated expansion of professional training and higher education for Ultra-Orthodox women, in contrast to the years in which these women worked almost exclusively in education. Ultra-Orthodox high schools have opened tracks for the study of additional subjects, and colleges and other higher-education programs aimed at this population now provide training in a variety of fields such as social work, law, business management, computer programming, communications, engineering, and psychology [43,44].

The rate at which Ultra-Orthodox women are employed in the areas in which they have trained is increasing rapidly, but it is still low, and most Ultra-Orthodox women prefer to complete their professional training in an Ultra-Orthodox environment [45]. Over the past 15 years, this population has experienced the sharpest increase in the employment rate of individuals between the ages of 25 and 64. In 2018, the employment rate stood at 76%, which is higher than the Organisation for Economic Co-operation and Development (OECD) average for female employment [46]. There has been an impressive increase in the proportion of Ultra-Orthodox women employed in the hi-tech sector, from less than 1% in the middle of the previous decade to more than 3% in 2018. Alongside this impressive increase, it is important to pay attention to the fact that many Ultra-Orthodox women are still employed in part-time positions (38.2%; [47]). Among those who are employed part-time, 55% work part-time due to their need to care for their families and households [46]. As they exit the educational field, which is within the Ultra-Orthodox space, and pursue high-quality work in the general space, Ultra-Orthodox women face several different kinds of barriers. Ideological barriers, the desire to maintain an Ultra-Orthodox lifestyle, and limited exposure to the world of secular content delineate the boundaries of the space into which Ultra-Orthodox women can integrate. Technical barriers to their integration into the general workforce include a lack of skills and professional knowledge in different fields. In addition, women who are managing large families have trouble filling in the gaps in their education and, therefore, tend to take part-time jobs. Structural barriers include hesitancy on the part of employers and the level of demand for female Ultra-Orthodox workers [43]. All of these factors make it difficult for these women to find high-quality work.

### 1.6. The Integration of Bedouin Arab Women into the Israeli Workforce

Since the 1990s, a number of factors have affected the growing proportion of Bedouin Arab women from southern Israel pursuing higher education. These include the foundation of the Center for Bedouin Arab Studies and the development of Ben-Gurion University of the Negev, which has offered full scholarships to Bedouin Arab women [48], and the increased awareness among Bedouin Arab families of the importance of education, in general, and higher education, in particular, as an important path for employment and financial independence. These factors have led to an understanding that women’s work will help raise their families’ standard of living, bringing it closer to that of the majority of Israeli society [4,49].

In contrast to the rapid improvement in education, the employment rates of Arab women have increased more slowly. In 2015, only 16% of the Bedouin Arab women in the Negev were in the workforce compared to employment rates of 64% for Jewish women in the Negev and 27% for all Arab women in Israel. Forty-eight percent of female Bedouin Arab workers were employed full-time (similar to the rate among all Arab women in Israel, in contrast to a rate of 51% for female Jewish workers), and 36% of female Bedouin Arab workers were employed on a part-time basis (as compared to 34% of Arab and Jewish women). Forty percent of Arab women reported that the main reason they were employed part-time was that they were unable to find full-time work compared to 14% of Jewish women [50].

Different factors have been identified as barriers to the participation of these women in the workforce, especially as regards professions that require academic training: labor market conditions and the number of available positions, lack of familiarity with Jewish-Israeli culture, low self-confidence, the language barrier [51], geographical distance, lack of daycare and public transportation, discrimination on the part of employers, mismatched expectations regarding employment conditions [52], religious norms, the traditional culture and society, and the division of gender roles [52,53]. All of these factors push women to capitulate to the pressures of work–home conflict [54]. These barriers also explain why the majority of college-educated Bedouin Arab women between the ages of 25 and 35, who hold degrees in different fields, choose to work in the fields of education and health only, which are seen as more feminine and allow them to work in their communities with hours that are convenient for them as mothers of young children [55]. The situation is even more complex in the unrecognized Bedouin villages of the Negev. The geographic, social, political, and economic isolation of these villages from the dominant labor market leads women from these villages who have acquired a higher education to return to their families and social groups, which are safe spaces. This situation requires women who have acquired different levels of education to support themselves in traditional roles [56].

It is important to note that Bedouin Arab women who hold a college degree are employed at a higher rate than those without a degree. Employment data for the years 2013–2015 show that 86% of employed Bedouin Arab women had a college degree, while only 10% of employed Bedouin Arab women did not have a college degree [4]. The large difference in the employment rates of Arab women with and without degrees is not only due to employers being persuaded to hire these women based on their academic credentials but also because these degrees point to the abilities of these women to successfully overcome social barriers in order to acquire higher education and enter the labor market [55].

In conclusion, Ultra-Orthodox society and Bedouin society are both undergoing a process of “academization” and an expansion of employment boundaries beyond the community, alongside a process of increasing their distinctiveness from their surroundings. There have even been incidences of strengthened opposition to individuals moving beyond the boundaries of the community based on a desire to preserve the community. While Ultra-Orthodox and Bedouin Arab women are both members of minority groups within Israeli society, they differ from one another in significant ways. Ultra-Orthodox women have been present in the Israeli workforce for two decades and have successfully integrated into influential positions in a range of fields [42]. In addition, the employment rate of college-educated Ultra-Orthodox women is about 90% [57]. The Ultra-Orthodox community has also developed a number of workplaces specifically for Ultra-Orthodox women beyond the field of education (some of which were developed with governmental assistance). In contrast, no similar significant initiatives have yet been developed for Bedouin Arab women.

The entrance of Bedouin Arab women into the Israeli workforce is a more recent phenomenon, and the employment rate of Bedouin Arab women is only 25%. However, among college-educated Bedouin Arab women, the employment rate is 70%. The range of fields in which educated Bedouin Arab women are employed is more limited than that of educated Ultra-Orthodox women and includes education, well-being, and healthcare [58].

### 1.7. Aims and Questions

In light of the significant changes—especially the integration of minorities—in different employment spaces around the world and in the Israeli economy, in particular, we wanted to evaluate whether and how SOC and a diversity climate in the workplace promote the advancement of college-educated women in workplaces outside their communities and strengthen their occupational health. We also sought to answer several secondary questions: Does a diversity climate help support personal SOC and, if so, how? What are the characteristics of a diversity climate? Finally, what differences do we see in the coping and optimal integration of college-educated Ultra-Orthodox and Bedouin women in the employment sphere?

## 2. Materials and Methods

### 2.1. Tools and Procedure

The materials upon which this qualitative work was based were gathered as part of an effort to evaluate the integration of Ultra-Orthodox and Bedouin Arab women into the labor market. The study included five focus groups of 16 women between the ages of 20 and 56. The participants in the focus groups had a variety of occupational backgrounds (e.g., accounting, management positions in local government, teaching, computer engineering, etc.). The Ultra-Orthodox participants included members of different Ultra-Orthodox streams (i.e., Lithuanian, Hassidic, Sephardic, and modern). Some of the participants worked in the public sector, and some worked in the private sector.

Focus groups are based on group interviews aimed at examining the personal ideas of the participants and acquiring a deep understanding of their feelings regarding the subject of interest [59]. In our focus groups, participants were asked to answer one main question, and, from that point, the discussion developed into a number of different directions. The discussions were mediated by a moderator in accordance with the dynamics that developed among the participants.

In January and February 2019, we conducted four focus groups with 16 women from the Ultra-Orthodox and Bedouin Arab communities. There were two focus groups of Ultra-Orthodox women, which each included five women, and three focus groups of Bedouin women, which each included three women. It was not easy to recruit women to participate in the focus groups due to the sensitive and personal topics. This was particularly true for the Bedouin women. These women were approached because they were educated and integrated into the Israeli space, two meaningful parameters for the definition of the target population for this study.

Two meetings with groups of Ultra-Orthodox women were held in Jerusalem, and two meetings with groups of Bedouin Arab women were held in Beersheba. Each group included a variety in terms of the participants’ ages, personal status, and fields of employment, but all of the participants were college-educated and defined themselves as members of those conservative communities.

A purposive sampling technique was used to recruit participants, as opposed to a random sampling technique. This is a method for sampling people who share a particular characteristic and is used to describe “politically important cases” [60]. These interviews were recorded and transcribed in full. The transcripts from the group interviews were subjected to comprehensive thematic analysis.

### 2.2. Data Analysis

These interviews were recorded and transcribed in their entirety. The transcripts were subjected to a thematic analysis. We chose to employ a narrative, phenomenological method, which allows the analysis of the experiences of the individual and the social reality from the subjective perspective of the study participants. The findings were analyzed using a diversity-climate lens [28] and salutogenic theory [34].

The main themes raised in the focus groups were categorized and analyzed to identify the employees’ perceptions and the resources that they have used to deal with stressful situations at work. We examined their perceptions regarding their employers’ creation and maintenance of a diversity climate within the organization and the significance of that climate for their own health and professional success.

## 3. Results

The findings of this study of the integration of Ultra-Orthodox and Bedouin Arab women into organizations are congruent with three of the four parameters of the diversity-climate approach. In practice, those parameters serve as a basis for strengthening SOC among the integrating workers. First, we will present an analysis of the statements made by the study participants, which were indicative of three main themes that parallel the three parameters of diversity climate (i.e., perceptions regarding policies and management processes within the organization, management activities that can lead to inclusion or exclusion, and level of comfort felt in interactions with members of other groups). Then, we will present our findings regarding how the diversity climate in different organizations supports SOC.

### 3.1. Perceptions Regarding the Diversity Policy of the Organization

The approach to diversity in the organizations in which the participants worked really stood out in all of the interviews. First of all, an organization that employs Ultra-Orthodox and Bedouin women is an organization that is open to accepting diverse candidates, including women from conservative backgrounds who are different from the majority of the organization’s workers. Second, all of the participants clearly viewed that fact as a feature of the diversity climate within the organizations in which they worked. This matter is a factor in the advancement of these women within their organizations, from the moment they are hired through their promotion, as they themselves noted.

Things are not perfect, with organizations varying in their treatment of minorities. It is also worth noting that, despite having been hired, some of the women mentioned a sense of not being accepted for a particular role or by clients or customers. As Bushra explained:

*Clients are not interested in a [female] Arab lawyer. In general, they think that a [male] Jewish lawyer is better and more knowledgeable. I applied to a lot of places. When I was hired (by the Public Defender’s office), everything changed. [I had] a permanent workplace; things began to be easy for me; I felt that all of my difficulties had disappeared and changed. A reliable, permanent place—much better. And in a field that I love: criminal law. When you work in a place like this, the cases come to you. Even my clients, who I worked with, changed their minds when they saw how professionally I worked and asked me to work for them privately. The Jews have the language and we don’t and so people think that they’re more professional.*

The Bedouin Arab women reported greater difficulty finding a job due to the language issue and ethnic issue and because they were seen as inferior to Jewish workers (as described above), which is intertwined with the complicated political situation in the country:

*During the interview and the admissions exams, they told me that the work was in Netivot. That’s a place with Jews and religious Jews and that deterred me a little. I took it as a challenge, to do my best to be professional. I went for it. I came from a place that was very well-known for its capabilities. During that period, there was the 2014 war with Gaza and I got married. I had a high-risk pregnancy and I kept working as hard as I could, out of a sense of responsibility and gratitude to my manager, because they really accepted me and taught me the course and paid for my training.*

*The thing that helped me to integrate me into the workplace was their warm support, that they waited for me and trusted my professionalism. We lack warm support in our personal lives. I found that at work. To be at peace with ourselves. Those are the things that connect me to our work world …When I worked in the Jewish sector, I found that they were forgiving of their workers and not suspicious of them.*

Aiman

### 3.2. Management Activities That May Lead to Inclusion or Exclusion

The second parameter of the diversity climate of an organization includes the organization’s actions with regard to the broad diversity of its workforce. The active measures taken by different organizations regarding the hiring of these women have several different aspects:

#### 3.2.1. Dealing with Tensions Related to Cultural Differences

The Ultra-Orthodox women noted the tension between their need to uphold religious principles at work and the differentness and different behavior of most of the workers in their workplace. On rare occasions, this tension prevented them from integrating properly. However, they reported that management generally worked to accommodate them and to find a variety of solutions.

*Today, there’s an understanding and a familiarity that comes from a more respectful place, [an understanding that] any change needs to be coordinated … The secular people understand that they don’t understand and so they’re respectful, keep a respectful distance, and, at the same time, they’re nice and interested and ready to be flexible. If there are disagreements, [they are] always [dealt with] through conversation and dialogue and very nice things happen.*

Dina

The Bedouin Arab women emphasized the cultural difference due to the ethnic difference:

*The [Jewish] culture has a good influence and led me to continue to work with them. When I chose to work with them, I was worried that they wouldn’t accept me. But, then I found that they accepted me and supported me, because you’re a minority among them and they look out for you.*

Rahma

The Ultra-Orthodox women and the Bedouin Arab women are both from communities that have high birthrates. In the Ultra-Orthodox community, the birthrate stands at 6.6% [43], and in the Bedouin community, the birthrate is 4.94% [3]. This cultural factor sometimes raises concerns among employers, who are worried about workers who might need to spend a lot of time on maternity leave and the negative effects that might have on the quality of their work. According to the participants, this concern is no longer as strong as it once was, and there is a consideration in this area. Additionally, the fact that these women were hired indicates that the management has already taken this issue into account. They also reported experiencing support and good relations.

*For, example, the third participant who carried on working hard and remained dedicated to her work during her pregnancy, because they supported her from the beginning and were attentive …*

Michal

Another issue shared by both groups of participants was that of religious fast days. Both religions have fasts at particular times during the year. Moslems have the month-long Ramadan fast. It is not easy to work while fasting, but the women reported that their employers were considerate of them in this area:

*I was on a panel and it was Ramadan and a fast and a war. I approached the manager and asked to be the first to present, so that I could break my fast and eat. Everyone looked at me like, ‘How can you ask for that?’ But, my manager supported me.*

Sabah

#### 3.2.2. Emotional and Instrumental Support

The Ultra-Orthodox women and the Bedouin Arab women both reported a sense of being valued that strengthened their own self-confidence and drove them to continue to work hard and invest in their work.

*I found support. They value me. As a Bedouin woman, they’re always telling me, ‘Good for you’. I have the confidence to stand opposite people when the language is strong with me. They share their thoughts and personal matters with me, their experiences. I was in a traffic accident and everyone came to visit me at home. They listen to me. My manager is always available for me, until late at night. He values my work and is sensitive with regard to my culture.*

Sara

The Ultra-Orthodox women also reported that they felt valued and respected. Both the Bedouin Arab women and the Ultra-Orthodox women reported a separation between their work and their leisure time and home lives. However, these boundaries were clearer among the Ultra-Orthodox women:

*They share about everything. My [female] boss shares about everything and she’s lovely. Everything’s out there, on the table. In terms of the staff, it’s a very good atmosphere. In my team, I brought in a team atmosphere; this was done purposefully. I built the team this way, so they’d know that I’m with them and they’ve responded in kind. They really respect me; I get a lot of love. I have friends at work, but they’re only friends at work, not in the afternoons.*

Ahuva

#### 3.2.3. Investment in Workers to Bridge Gaps

Since these women are members of culturally distinct minority groups, there are sometimes gaps between them and the other workers in their organizations. One example involves mastery of the English language, as most of the schools in their communities do not provide their students with a strong proficiency in that language. Therefore, as noted by the participants, organizations sometimes send these women to courses to help bridge these knowledge and language gaps.

This is how one Ultra-Orthodox woman, named Michal, described her own growth and her employer’s investment in her:

*I’m developing and learning all the time … I feel that they value my abilities … I was in a place that was thriving, so I thrived and grew, too.*

### 3.3. Level of Comfort Felt in Interactions with Members of Other Groups

The participants’ statements revealed a clear picture of a sense of comfort in the workplace and positive personal relations with colleagues of different backgrounds. As the women told us:

Michal: *“I’d like to preserve the atmosphere that’s been created at work”*.

Sara: *“The managers and the workers work together and respect each other”.*

That said, the participants also described complicated and uncomfortable situations that had arisen over the years between workers and management. Whereas the Ultra-Orthodox women felt discomfort related to religious issues, the Bedouin Arab women mentioned difficulties related to language and knowledge gaps, as well as discomfort during periods of political tension or in nationalistic contexts that arose in their interactions with their colleagues. The Ultra-Orthodox women were very satisfied and felt respected when their organizations met their religious needs. As Rachel told us:

*I work in a secular place. They’re very respectful; I have no complaints about them. If somebody needs food with a particular kashrut certification, they always respect that. They respect each person’s level of religiosity … there’s a lot of professionalism there.*

*Partial integration, also on a daily basis. I work with Ultra-Orthodox people and with people who are not Ultra-Orthodox, who have come to work with the Ultra-Orthodox. There are workplace dilemmas regarding men ….*

The religious dilemmas do receive an appropriate response, and this creates a bond between the Ultra-Orthodox women and their colleagues. The solutions that have been found have led the Ultra-Orthodox women to feel comfortable expressing themselves. As Miri explained:

*I do feel that they cooperate and share and, if they don’t, I take the initiative. There was a conference that I wasn’t invited to, that I was interested in. I said, ‘Next time, you’ll invite me’.*

Thanks to the sense of being accepted from the start, Miri felt comfortable making that statement. This behavior is not typical of members of minority groups, who tend to wait to be invited or to share due to a fear that they are not wanted.

As noted above, the Bedouin Arab women sometimes felt uncomfortable due to the ethnic-identity issue. However, they reported that, over time, that issue became less acute, and their interactions in the workplace were based on relationships characterized by trust and respect:

*I sometimes went to places related to national security. I was responsible for hazardous materials and went for inspections. At first, they were confused because of my name and surprised by my clothing and the headscarf that I wear. They always looked and checked whether or not I was serious. But, after a few times, the people trusted me and asked the office that sent me to send ‘the Bedouin woman’ because they saw that I was professional.*

Sabah

### 3.4. A Diversity Climate Enhanced the Sense of Coherence among the College-Educated Women

As described above, the Ultra-Orthodox women and the Bedouin Arab women reported positive feelings regarding their integration into different organizations. Diversity climate appears to play a critical role in the integration of these minority women, and there is an interaction between instilling such values and support for SOC. SOC has three main components: comprehensibility, manageability, and meaningfulness. All three of those components were clearly observed in the focus groups with both groups of women.

Comprehensibility is strengthened by the promotion of a sense of confidence, and a safe and respectful work environment strengthens its social aspects Aiman described this well:

*[I] feel proud. Everybody’s an expert from the south and everybody’s professional. I work with skilled people who have magnificent abilities. I very much feel that I belong. Today, I’m one of the better people in the non-profit organization and I feel special. When you’re in a place where there’s support and expertise, you get used to good things.*

The manageability component also came across clearly in the focus groups. The sense of manageability is strengthened when people feel that their needs are being met and that they have the tools they need to deal with different situations. Manageability is associated with experiences of self-efficacy, a balance between heavy workloads and less heavy workloads, the achievement of goals and the recognition of those achievements, and the acquisition of tools and experience.

*Today, I feel confident in the field in which I work. I didn’t break and I got here through my own efforts… really empowers the personality and the soul. I don’t need to tell the people around me what I’ve done. Our obligation is to accept ourselves as we are with the abilities that we have.*

Miri

After describing their initial acceptance into their different workplaces and sharing their stories of those experiences, the women noted that they were each in a workplace that was quite meaningful for them. Meaningfulness is promoted by the feeling that one can influence decisions, be creative in one’s work, and, at the same time, contribute to the support of one’s family.

*I give more than 100% at work, because I feel that I’m a representative and I have an obligation to my community.*

Rachel

This statement can be grouped together with other statements made by the participants regarding the strengthening of the sense of meaningfulness over the years they had worked in their organizations, increased self-confidence, and health and calmness in the workplace.

## 4. Discussion

Different theoretical frameworks have been used to examine the integration of members of cultural minority groups in the workforce. In this work, we focused on factors that promote the occupational health and wellbeing of college-educated ultra-Orthodox and Bedouin Arab women in the workforce from their own points of view. This work was based on two theoretical frameworks: salutogenic theory and the diversity-climate approach. These theoretical frameworks are part of a positive, proactive health trend, which has been examined at both the individual and the organizational levels and which tries to identify qualities that preserve and promote personal, social, and environmental resources, to help those who have joined organizations cope with the challenges in their environments.

In our focus groups, the woman spoke about their experiences in a number of different workplaces and discussed their employment histories, comparing less inclusive and more inclusive places in which they had worked. In Section 3 of this work, we chose to include descriptions of the workplaces that were more inclusive and which used a variety of tools to integrate these women.

We found evidence of the three main components of a diversity climate in the participants’ statements, namely: (a) perceptions regarding policy and management processes, (b) management actions that could lead to the inclusion or exclusion of members of minority groups, and (c) the level of comfort experienced in interactions with members of other groups. Interestingly, the fourth dimension—the personal dimension that relates to an individual’s perceptions regarding the importance of diversity in work teams and the organization as a whole—was not mentioned by the participants.

We also found that the three components of SOC (i.e., comprehensibility, manageability, and meaningfulness) are nurtured by a diversity climate and the degree to which such a climate is implemented with regard to the integrating workers. These findings show that a diversity climate contributes to and strengthens personal SOC, with an emphasis on meaningfulness, which contributes to and promotes the personal health of the integrated workers.

The participants’ reports confirm the perception that diversity climate is a conceptual environmental resource that helps workers to strengthen their own emotional resources. They also show that an individual’s experience of a positive organizational climate is related to a high level of salutogenic functioning [30].

Parameters related to hope, optimism, self-efficacy, and resilience came up repeatedly in the focus groups. They appeared in the participants’ descriptions as factors that supported their sense that they played essential roles in their organizations [15]. When personal resources are empowered, the individual is well-situated to deal with pressures. Those resources are a source of tremendous energy and motivation in the encounter with work demands [23]. Since individuals spend most of their time at work, their health at work is an inseparable part of their general health [24].

Management that works to create and support a diversity climate will respect the different perspectives and ideas of each staff member, communicate openly, be accessible, and acknowledge and express concern for everyone’s needs, expectations, and feelings [25]. Workers’ dedication to their tasks increases when they feel more comfortable suggesting initiatives and working hard to carry them out.

### Study Limitations and Avenues for Further Research

The study has several limitations. First, we propose conducting a more comprehensive study that will also include employers and will reflect the integration process from their perspective. Second, it would be worthwhile to expand the number of interviewees or to distribute a comprehensive questionnaire to a larger population, to confirm the major themes that emerged from this study. A large number of participants can provide a broad picture. Third, it would also be worthwhile to conduct a comparative study of the integration of men and women from these communities. Such a study could reveal interesting differences that could hone our understanding of the issue and shed light on the communities participating in the study, with an emphasis on the gender aspect.

## 5. Conclusions

Our findings indicate that almost all of the organizational dimensions discussed in this work, as well as the support given by managers, affect the different components of SOC: comprehensibility (i.e., individuals’ understanding of the bigger picture), manageability (i.e., their ability to cope), and meaningfulness (i.e., their emotional commitment to their work). The examined factors also affect the ability of individuals to see a connection between their own behaviors and different outcomes, the degree of personal control and freedom that they can exercise at work, and their subsequent feelings of empowerment. A workplace in which individuals feel comfortable contributing information and making suggestions will also provide fertile ground for effective collaboration among colleagues. Here, we demonstrate how salutogenic theory explains how a diversity climate in the workplace can facilitate the integration of college-educated women from socially conservative communities.

The findings of the study can be used to help the Ultra-Orthodox community, the Bedouin Arab community, and the Israeli economy to improve the integration of these women into the general workforce. First, we recommend developing programs to address the integration challenges and conflicts faced by members of the Ultra-Orthodox and Bedouin Arab communities, starting at the school stage. These programs could help to reduce stress, enhance workplace integration, and promote the peace of mind and mental health of those who integrate. It is also worth considering the development and expansion of diversity programs for employers to teach employers about the experiences of those who integrate and the struggles of minorities in the job market. Expanding employers’ knowledge of these issues could help to improve the recruitment and retention of employees, benefiting both employers and employees who are members of minority groups.

## Data Availability

The raw data supporting the conclusions of this article will be made available by the authors without undue reservation.
